# Supplementary material for: Comprehensive Analysis Identifies THEMIS2 as a Potential Prognostic and Immunological Biomarker in Glioblastoma
Source: Cells. 2025 Jan 7;14(2):66. doi: 10.3390/cells14020066 (PMC11764009; doi:10.3390/cells14020066)

Supplementary Figure S1. Macrophage Markers

Markers used for cell annotation during macrophage identification, showing expression patterns and providing evidence for macrophage classification in the single-cell dataset.

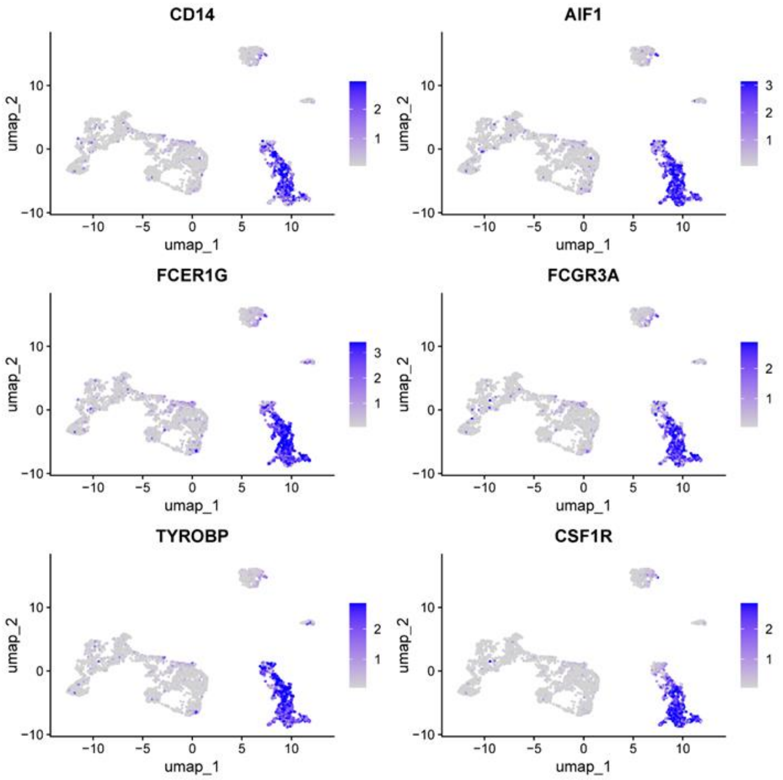

Supplementary Figure S2. Immune Infiltration and THEMIS2 Correlation in CGGA dataset.

(A, B) MCP-counter and xCell analyses indicate increased infiltration of monocytes, dendritic cells, and B cells with high THEMIS2 expression in the CGGA dataset. (C) ssGSEA analysis demonstrates a positive correlation between THEMIS2 expression and infiltration of multiple immune cell types in the CGGA dataset.

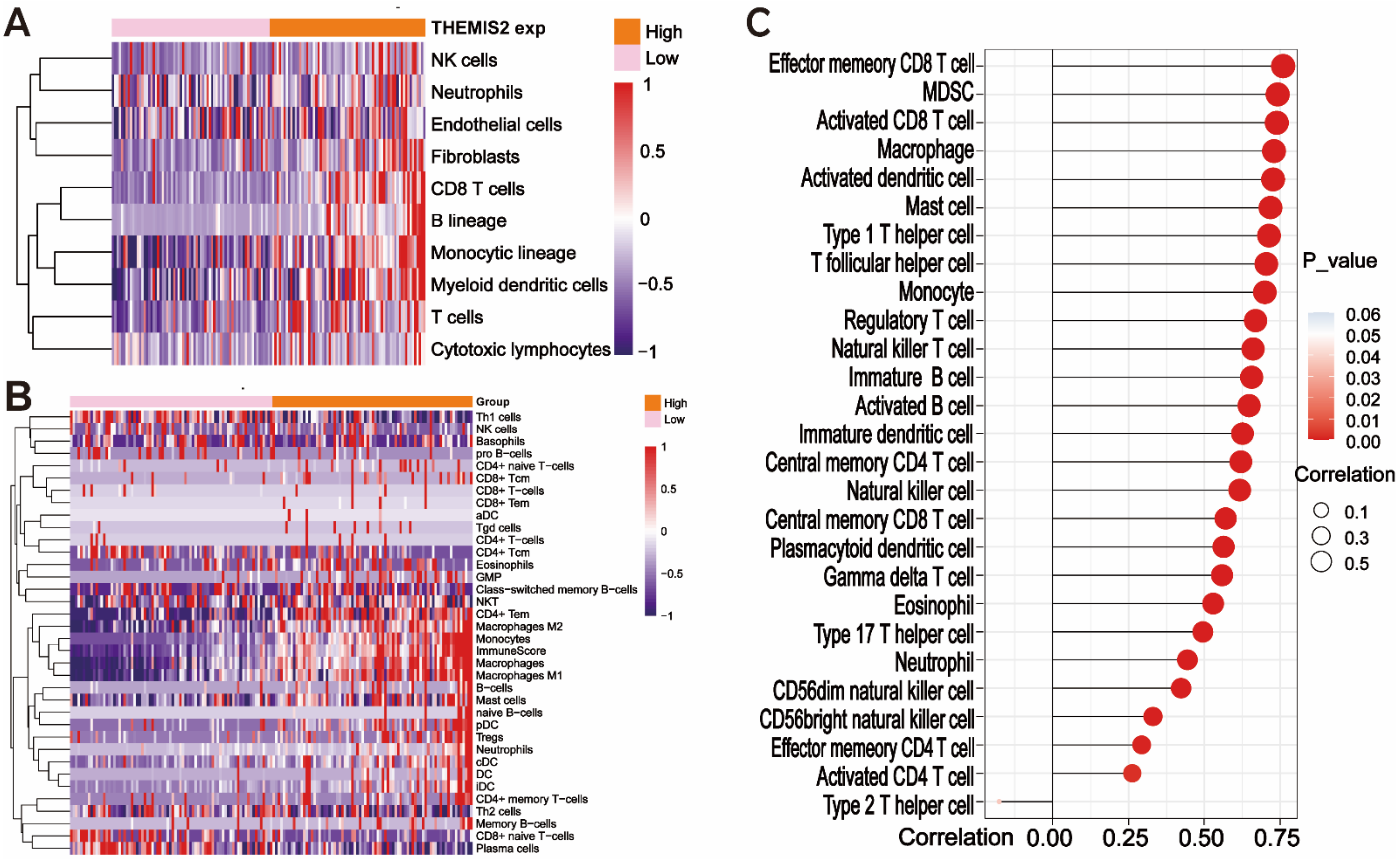

Supplementary Figure S3. ***THEMIS2*** **Expression, Immune Microenvironment, and Immune Checkpoints.**

(A - C) Correlation of *THEMIS2* with ESTIMATEScore, ImmuneScore, and StromalScore in CGGA, indicating positive correlations. (D) Correlation of *THEMIS2* with common immune checkpoint molecules in CGGA, suggesting a role in an immunosuppressive microenvironment.

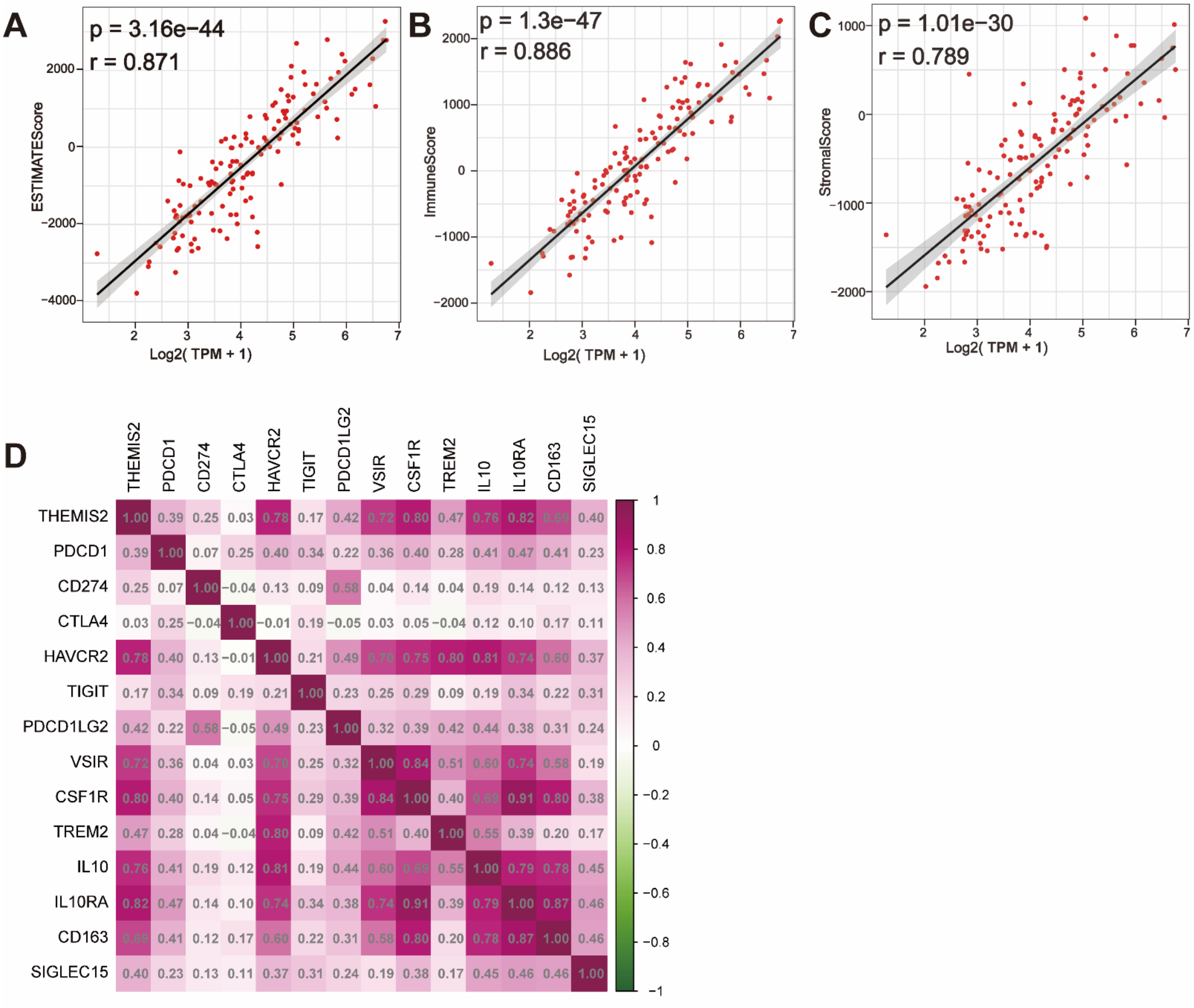

Supplementary Figure S4. ***THEMIS2*** **Expression and Prognosis in MGMT-Methylated Glioblastoma**

(A) In the CGGA dataset, high THEMIS2 expression is associated with significantly poorer overall survival in patients with MGMT-methylated glioblastoma. (B-D) No statistically significant survival differences are observed in other subgroups, including (B) CGGA MGMT-unmethylated, (C) TCGA MGMT-methylated, and (D) TCGA MGMT-unmethylated patients, as shown by Kaplan-Meier survival curves.

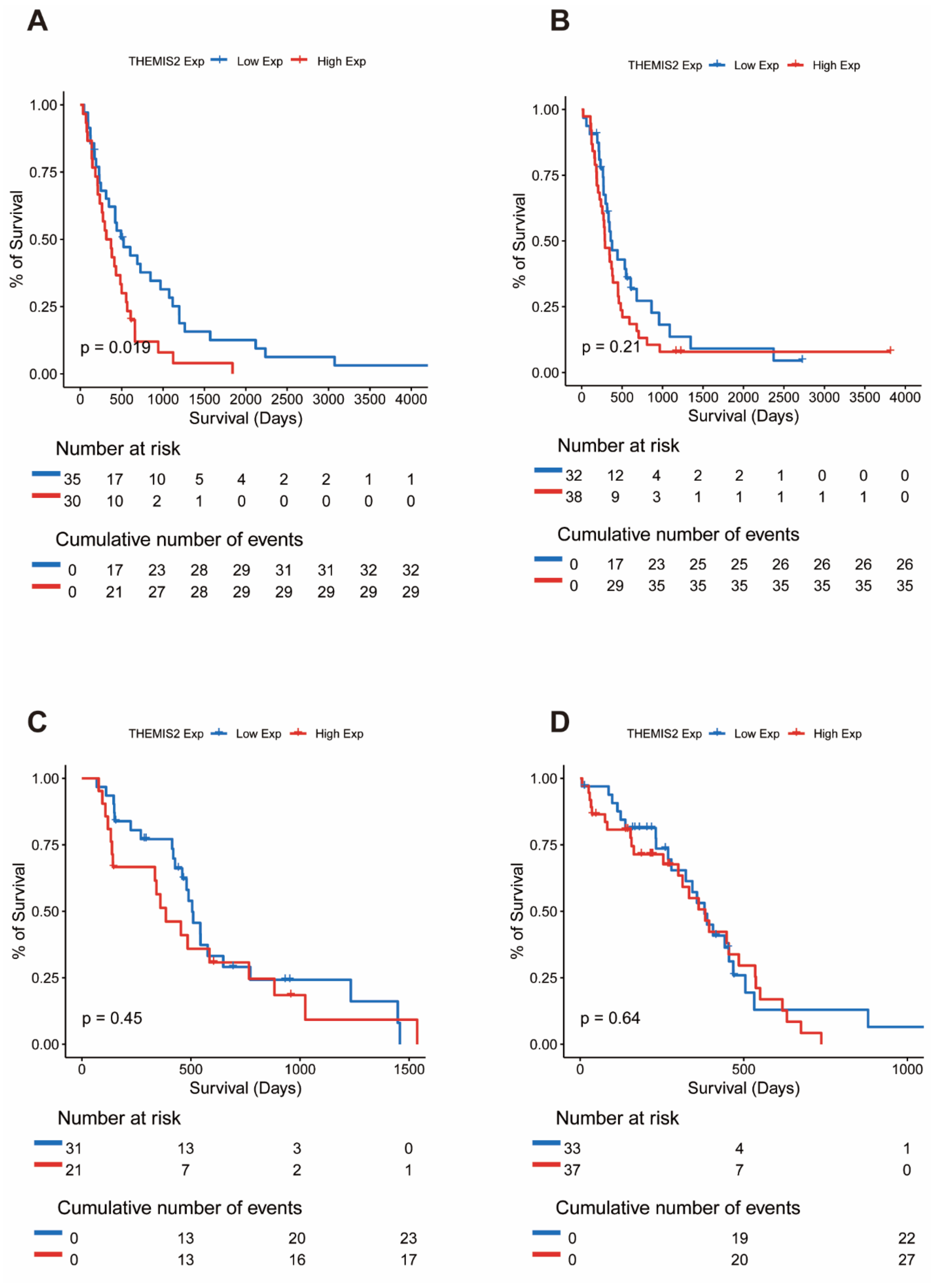

Supplement: Supplementary file 1 [file cells-14-00066-s001.zip › cells-3366170-supplementary.pdf]
